# Prevalence of Diabetes and Associated Risk Factors among a Group of Prisoners in the Yaoundé Central Prison

**DOI:** 10.1155/2020/5016327

**Published:** 2020-01-24

**Authors:** Sylvain Raoul Simeni Njonnou, Jérôme Boombhi, Martine Claude Etoa Etoga, Aimée Tiodoung Timnou, Ahmadou Musa Jingi, Kevin Nkem Efon, Esther Astrid Mbono Samba Eloumba, Marie-Josiane Ntsama Essomba, Odette Kengni Kebiwo, Alice Ninon Tsitsol Meke, Stéphane Talbit Ndjonya, Mesmin Dehayem Yefou, Eugène Sobngwi

**Affiliations:** ^1^Department of Internal Medicine and Specialties, Faculty of Medicine and Biomedical Sciences, University of Yaoundé I, Yaoundé, Cameroon; ^2^Yaoundé General Hospital, Yaoundé, Cameroon; ^3^Department of Clinical Sciences, Faculty of Medicine and Pharmaceutical Sciences, University of Douala, Douala, Cameroon; ^4^Yaoundé Central Hospital, Yaoundé, Cameroon; ^5^Yaoundé Central Prison, Yaoundé, Cameroon

## Abstract

**Background:**

Diabetes is a public health problem worldwide, associated with increased morbidity and mortality. According to the International Diabetes Federation (IDF) 2017 data, around 425 million people worldwide suffer from diabetes. This number is expected to increase to 629 million in 2045. Various occidental studies reported the increased prevalence and lower control of diabetes among prisoners. However, there is no data on the characteristics of inmates with diabetes in sub-Saharan Africa.

**Methods:**

A cross-sectional study among incarcerated detainees from the Yaoundé Central Prison was conducted from January to July 2017. Diabetes was defined according to the American Diabetes Association (ADA) criteria. Analyzed variables included phenotypic characteristics, lifestyle, the reason for detention, the sentence severity, and the length of detention.

**Results:**

We recruited 437 inmates (344 men) with an average age of 37.0 (95% CI: 35.9-38.3) years. The most frequent age group was 20 to 39 years with 281 (64.7%) inmates, and the mean prison stay was 29.1 (95% CI: 25.7-32.8) months. The prevalence of diabetes in the Yaoundé Central Prison was 9.4%. The main cardiovascular risk factors were a sedentary lifestyle (91.1%), hypertension (39.6%), smoking (31.6%), and alcohol consumption (28.1%). Hypertension (*p* = 0.005), obesity (*p* = 0.005), obesity (*p* = 0.005), obesity (*p* = 0.005), obesity (*p* = 0.005), obesity (*p* = 0.005), obesity (

**Conclusion:**

Diabetes prevalence in the Yaoundé Central Prison was high, at 9.4%, compared to that in the general population. It was associated with other classical cardiovascular risk factors and factors linked to the sentence (minor and major crimes). This trial is registered with CE00617/CRERSHC/2016.

## 1. Background

Many studies reported an increase in the carceral population worldwide, estimated at almost 11 million. This number is probably underestimated due to false reports or the unavailability of many of them [1]. Diabetes is a major public health problem in the world, with a global prevalence estimated at around 8.8% of the world population (425 million people), according to the IDF 2017 data [2]. This number is expected to increase to 629 million in 2045. Moreover, diabetes is associated with severe morbidity and mortality: 4 million of death in 2017 were diabetes-related and the cardiovascular complications are common [3–12]. The diabetes prevalence in the United States of America (USA) was estimated at 9.3% (30.2 million) in 2017. It is lower in Africa with 4.2% but probably underestimated because data are lacking [2].

Racial disparities in diabetes prevalence and care are well recognized, with increased prevalence among the Afro-American population and Hispanic population compared to the white population [13–16]. Diabetes remains one of the principal cardiovascular risk factors in Africa [17]. The prevalence of diabetes in Africa is the lowest (4.2%) compared to other continents, but the prevalence and the burden of this disease are rising quickly in Africa [18, 19]. Uncontrolled urbanization and major changes in lifestyle seemed to drive this burden [20, 21]. In Cameroon, diabetes prevalence was estimated at around 6% in 2018 [2, 22]. This prevalence is increasing in the general population, rising from 2.0% in 1999 to 4.7% in 2002 and 5.8% in 2018 [22–24]. There is a regional disparity between rural and urban areas, with a rural prevalence of diabetes lower than the urban one but rising with time [23–25]. Diabetes prevalence also seems to be increased in particular groups: patients with stroke (12.8%) and patients with end-stage renal disease (15.9%) [26, 27].

Glucose impairment seems to be associated with older age, lifestyle modification, inflammatory status, and the presence of other cardiovascular risk factors [24, 28, 29]. Other factors are also described: heredity, genetic predisposition, the influence of the environment, and lifestyles such as salt consumption and sedentary lifestyle. Nevertheless, the contribution of stress (professional, family, and social) is far from unanimous. It can be involved in the onset of diabetes or completely decompensate a well-controlled disease. Prison is known to be a stressful environment [30]. This condition can reveal or disturb the disease [31]. However, several studies in western countries showed similar or reduced diabetes prevalence among prisoners [32–34]. This prevalence could be higher among a particular group of prisoners, especially those over 50 years [35]. In Cameroon, previous studies reported promiscuity, increased carceral population, and high prevalence of obesity and hypertension among prisoners [36, 37]. It was therefore important for us to assess the prevalence of diabetes among prisoners and identify the associated factors.

## 2. Methods

### 2.1. Study Design and Setting

A cross-sectional study was conducted among Yaoundé Central Prison inmates from January to July 2017. This prison is located in Yaoundé, the city capital of Cameroon (sub-Saharan Africa), with a population of 2 million individuals. It is the main prison of this city. Although the prison was initially built for 800 prisoners, the carceral population has been evaluated at 4859 inmates in July 2017. The prison is organized in 206 rooms divided into 13 quarters. Living conditions in the Yaoundé Central Prison are bad: promiscuity and malnutrition rate are high among prisoners and bedding condition is poor (most of the prisoners sleep on a mattress laid on the floor). Clinical follow-up on prisoners is poor (there are only two doctors for managing all prisoners), and physical activities are rare [38].

### 2.2. Variables and Measurements

All inmates were approached regardless of the in-prison stay, the sentence, or the in-prison location. Clear information was given to all inmates about the study and their participation. All consenting inmates who came at the in-prison clinic were included in the study. We excluded all subjects who did not fast, who did not sign a consent, and who were not seen at the in-prison clinic at the second passage. The second passage was useful for collecting and confirming an inmate's information. The lack of data and the not fasting situation were the most exclusion criteria.

#### 2.2.1. Procedures

Trained medical personnel conducted the screening at the in-prison clinic. The participants underwent a face-to-face interview. All consenting participants were seen twice at a 1-month interval. Using a standardized questionnaire, data were collected on demographics, incarceration characteristics (duration, the severity of condemnation), smoking habits and alcohol consumption, diabetes and hypertension history including drug treatment and complications, and other cardiovascular risk factors.

**Figure 1:**
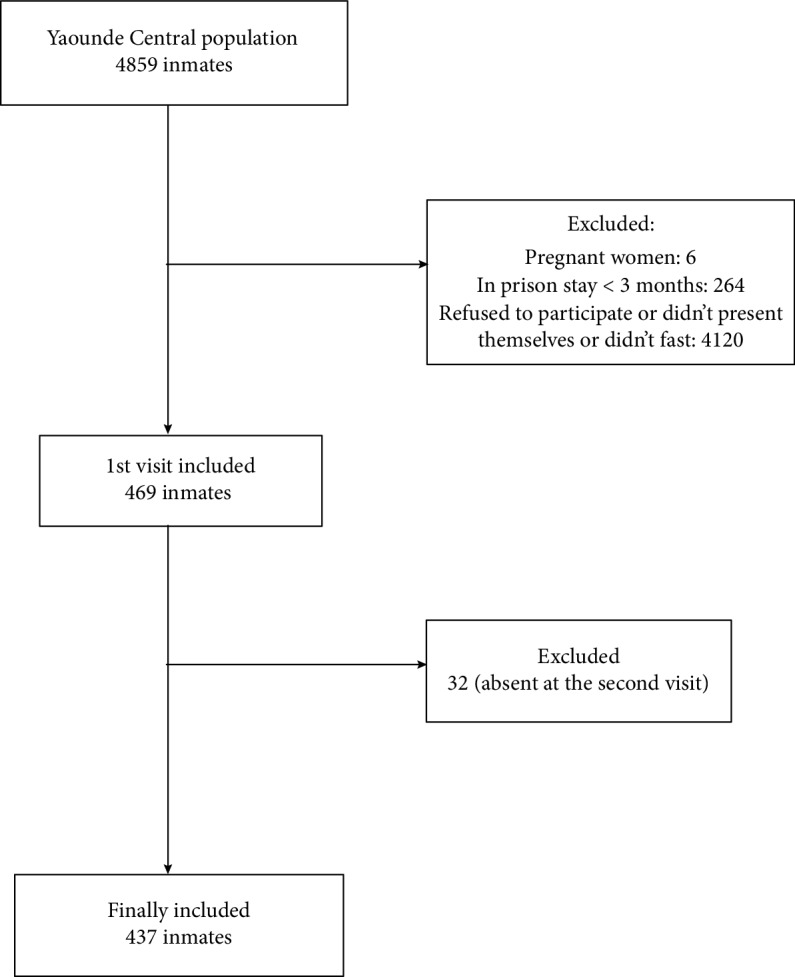
Patient flow chart.

All participants were subjected to a physical examination. It included BP and anthropometric measurements. The BP (in mmHg) was measured in each arm using a standard adult arm cuff of an aneroid manometer (OMRON) with the patient's arm supported and at least 10 min after the patient was rested in a sitting position. The measurement was repeated 2 minutes later. The average of the two measurements obtained in each arm was taken as the patient's blood pressure. Mean BP of both visits was registered. Height (in m) measurement was done to the nearest 0.01 m using a wooden platform and a height ruler, without shoes, feet together, arms by the sides, and in an erect position on the wooden platform footrest. Weight (in kg) was measured to the nearest 0.5 kg using a weight scale with the patient wearing only light clothing. Waist circumference (WC) was measured in a horizontal plane midway between the inferior margin of the ribs and the superior border of the iliac crest with the patient standing erect and breathing normally. A nonextensible measuring tape graduated in centimeters was used for the measurement. The body mass index was calculated as weight (kg)/height (m) × height (m). Fasting capillary blood sugar was measured using a OneTouch® brand glucometer. The selection process is summarized in Figure 1.

### 2.3. Definitions

Patients were declared diabetic according to the following criteria defined by the American Diabetes Association: a fasting plasma glucose (FPG) level ≥ 126 mg/dL (7.0 mmol/L) on two separate occasions: a random blood glucose ≥ 200 mg/dL (11.1 mmol/L) and a two-hour plasma glucose concentration ≥ 200 mg/dL (11.1 mmol/L) after 75 g anhydrous glucose in an oral glucose tolerance test (OGTT) [39]. People with a previous diagnosis or treatment for diabetes were also considered diabetic. Systolic BP ≥ 140 mmHgand/or diastolic BP ≥ 90 mmHg defined hypertension. Obesity was defined as a BMI of ≥30 kg/m^2^, and overweight was defined as a BMI between 25 and 29.9. A sedentary lifestyle was defined as the absence of any physical activity (absence of at least 3 walking episodes of 45 min in a week).

Waist circumference > 94 cm in men or 80 cm in women was high. Excessive alcohol consumption was based on intake either more than 3 (2 for women) standard glasses of wine per day or more than 10 (5 for women) local beers per week. Traditional alcohol beverage was not assessed. Current smoking was defined by a consumption of at least one cigarette per day.

A monthly income of less than 86.3 $US defined low social class. Other social classes were classified into middle (by income between 86.3 and 258.9 $US) and high (by an income above this amount).

Cameroon's penal code (2016 version) classified the reason for detention into felonies or crimes (an infraction punishable with death or with loss of liberty for a maximum of more than 10 years and fine where the law so provides) and offense (an infraction punishable with loss of liberty or with fine, where the loss of liberty may be for more than 10 days but not for more than 10 years and the fine more than 43.2 $US). The crimes may be subdivided into minor (use or selling of drugs, the unauthorized use of a weapon, or robbery) and major (homicide, rape, or embezzlement of public funds) crimes [40].

Sentence severity was subdivided into short (<5 years), average (5 to less than 15 years), and severe (>15 years). In the same way, an incarceration length < 5 years, between 5 and 15 years, and more than 15 years was classified as short, average, and high, respectively.

### 2.4. Sample Size and Statistical Analysis

The sample size was calculated using Lorenz's formula (StatCalc of EPI Info software). Using the national prevalence of 5.8% in Cameroon, with an 80% power to detect significant associations or differences and a 5% accepted margin of error, the minimal sample size estimate was 84 participants.

Data were analyzed using the Statistical Package for the Social Sciences (SSPS Inc., Chicago, Illinois, USA) v.20.0 and EPI-INFO v.3.5 software. Quantitative data are presented as median ± confidence interval (CI), and qualitative data are presented as frequencies and percentages. Group comparisons were performed with chi-square tests and equivalents for qualitative variables and the Student *t*-test and analysis of variance (ANOVA) for quantitative variables. Factors associated with diabetes in univariate analysis were entered into a multivariate logistic regression. Results were considered statistically significant for *p* values < 0.05.

## 3. Results

### 3.1. Participants

**Figure 2:**
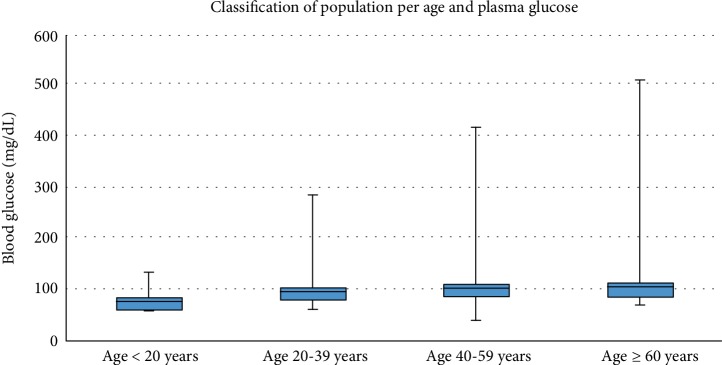
Population classification by age and mean plasma glucose.

We included 437 inmates, mostly from male gender (344, 78.7%), with a median age of 37 (95% CI: 35.9–38.3) years. The most frequent age group was 20 to 39 years with 281 (64.7%) inmates (Figure 2). The median prison stay was 29.1 (95% CI: 25.7-32.8) months. Among them, 41 (9.4%) inmates were diabetic. Median blood sugar was 90 (95% CI: 0.79-1.07) mg/dL. Median blood glucose increased with age, from 0.75 for the <20 years group to 1.21 for the ≥60 years group (*p* < 0.001) (Figure 2).

### 3.2. Main Data

**Figure 3:**
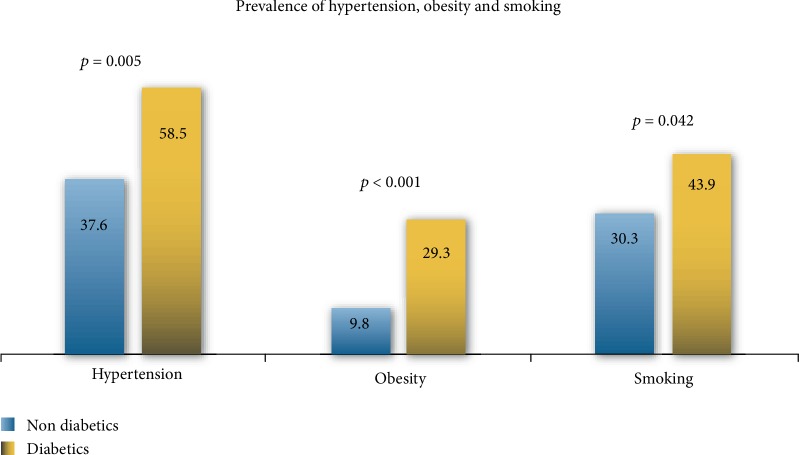
Comparison of diabetic and nondiabetic patients according to hypertension, obesity, and smoking.

Sedentary lifestyle (91.1%), smoking (31.6%), hypertension (39.6%), and alcohol (28.1%) were the main cardiovascular risk factors among inmates. 24 (58.5%) diabetic inmates were also hypertensive. The cardiovascular risk factors are presented in Table 1. Hypertension (*p* = 0.005), obesity (*p* < 0.001), smoking (*p* = 0.042), and sedentary lifestyle (*p* = 0.039) were associated with diabetes (Figure 3).

Inmates from the low social class were the most frequent, 241 (55.1%) inmates, followed by those from the high social class (122, 28.1%). Among those inmates, 226 (51.7%) of them were accused guilty, 94 (21.5%) were condemned for less than 5 years, 74 (16.9%) were condemned for 5 to 15 years, and 43 (9.8%) were condemned for more than 15 years.

Among detention variables, only major crime (*p* = 0.007) and minor crime (*p* = 0.003) were associated with diabetes. Neither social class nor sentence length nor sentence severity was associated with diabetes in univariate analysis. In multivariate analysis, only obesity was associated with diabetes among inmates (Table 2).

## 4. Discussion

This cross-sectional study is aimed at assessing the prevalence of diabetes and the associated risk factors among a group of prisoners in a sub-Saharan African setting. This study represents the first published data analysis of diabetes in a specific population of inmates in Africa.

Evidence from western countries shows that noncommunicable disease (NCD) is a public health problem in prison [34, 35, 41–45]. Recent studies in Africa showed an increased prevalence of NCD among prisoners [44, 45]. Diabetes prevalence among prisoners is still poorly known in Africa. However, there is no data in Africa and particularly in Cameroon for its prevalence and risk factors.

This study should be interpreted considering some limitations. As we carried out this study in only one prison of Cameroon and an urban area, the findings could not be extrapolated to others. Another important limitation was the diagnostic mean: most of the diabetes diagnosis was made based on fasting capillary glucose, and OGTT and glycated hemoglobin were not performed. The other limitations were the population size (small compared to other studies on the topic) and the fact that the study relied on a population census that was not designed to explicitly measure the effect of exposure to incarceration (length). Our capacity to make causal inferences is limited by the cross-sectional design of the study.

Diabetes prevalence was 9.4% in the Yaoundé Central Prison. This is higher than the national prevalence of 5.8% [2, 22]. Diabetes prevalence among prisoners used to be the same or lower to the national prevalence [32, 34, 44, 46, 47]. This increased diabetes could be related to poor feeding conditions and sedentary lifestyle. It has thus been proven that improvement of diet and physical activity could improve diabetes control or metabolic syndrome among prisoners [48, 49]. However, considering the high prevalence of CVRF in this population, the inappropriate diet, and the prevalence of infectious conditions, this prevalence seems to be reliable [37]. Furthermore, Kamdem et al. reported increased overweight/obesity and impaired fasting blood glucose in teenagers, the same as Mandob who reported a prediabetes prevalence of 15.3% [50, 51]. It is therefore conceivable that such a population put in this environment could develop diabetes. Sedentary lifestyle, hypertension, smoking, and alcohol consumption were the main CVRFs in this population. This was similar to Herbert's, Munday's, Maimela's, and Sabir's findings, showing low obesity and physical activity among prisoners, while smoking and alcohol consumption are increased [35, 43–45]. As in the normal population, plasma glucose levels turn to increase with age [52].

Detention variables associated with diabetes were only the reason for detention. Major crime (*p* = 0.007) and minor crime (*p* = 0.003) and middle social class (*p* = 0.001) were associated with diabetes. This could be because major and minor crimes included financial crimes or fraud which in the Cameroonian setting is used to be done by people from a certain age and probably with diabetes before prison.

Preventive strategies need to be implemented regarding the high prevalence of diabetes and CVRFs among prisoners. Importance should be given on screening for diabetes, hypertension, and other CVRFs. Lifestyle modification (alcohol and smoking cessation, appropriate diet, and increased physical activity) should be in the foreground. This also means increasing the number of caregivers and making health promotion among prisoners.

## 5. Conclusion

Although this study is the first of its kind in Cameroon and despite its limitations, it shows a high prevalence of diabetes in the Yaoundé Central Prison. Risk factors for diabetes among prisoners included detention variables such as the sentence for a major or minor crime and classical CVRFs such as sedentary lifestyle, hypertension, smoking, and obesity. Due to the high prevalence of diabetes among this group of prisoners, there is a need to implement preventive strategies for diabetes in prison.

## Figures and Tables

**Table 1:** Risk factors associated with diabetes among inmates in univariate analysis.

Variables	Overall	Diabetes+, *n* (%)	Diabetes-, *n* (%)	OR (95% CI)	*p* value
Sex					
Male	341 (100)	35 (10.3)	306 (89.7)	1.7 (0.7–4.2)	0.118
Female	96 (100)	6 (6.3)	90 (93.7)		
Age (years)					
<20	6 (100)	1 (2.4)	5 (1.3)	1.9 (0.2–17.1)	0.448
20-39	281 (100)	23 (57.5)	258 (65.5)	0.7 (0.4–1.3)	0.160
40-59	109 (100)	13 (31.7)	96 (24.2)	1.4 (0.7–2.9)	0.150
>60	41 (100)	4 (9.8)	37 (9.3)	1.0 (0.3–3.1)	0.552
Social class					
Low social class	241 (100)	22 (53.7)	219 (55.3)	0.9 (0.5–1.8)	0.419
Middle social class	74 (100)	6 (14.6)	68 (17.2)	0.8 (0.3–2.0)	0.355
High social class	122 (100)	13 (32.5)	109 (27.7)	1.3 (0.6–2.5)	0.258
Hypertension	173 (100)	24 (58.5)	149 (37.6)	2.3 (1.2–4.5)	0.005
Obesity	51 (100)	12 (29.3)	39 (9.8)	3.8 (1.8–8.0)	<0.001
Smoking	138 (100)	18 (43.9)	120 (30.3)	1.8 (0.9–3.4)	0.042
Alcohol consumption	123 (100)	14 (34.1)	109 (27.5)	1.4 (0.7–2.7)	0.187
Sedentary lifestyle	398 (100)	34 (82.9)	364 (91.9)	0.4 (0.2–1.0)	0.039
Reason of detention					
Offense	250 (100)	22 (53.7)	228 (57.6)	0.8 (0.4–1.6)	0.315
Minor crimes	67 (100)	1 (2.4)	66 (16.9)	0.1 (0.01–0.9)	0.003
Major crimes	120 (100)	18 (46.2)	102 (26.7)	2.3 (1.2–4.6)	<0.001
Length of incarceration					
Low	387 (100)	33 (80.5)	354 (89.4)	0.5 (0.2–1.2)	0.055
Average	41 (100)	7 (17.1)	34 (8.6)	2.2 (0.9–5.3)	0.051
Long	9 (100)	1 (2.4)	8 (2)	1.2 (0.1–9.4)	0.591
Sentence severity					
Short sentence	94 (100)	9 (22)	85 (21.5)	1.0 (0.5–2.2)	0.460
Average sentence	74 (100)	5 (12.2)	69 (17.4)	0.6 (0.2–1.7)	0.206
Severe sentence	43 (100)	7 (17.1)	36 (9.1)	2.0 (0.8–4.9)	0.064

**Table 2:** Risk factors associated with hypertension in multivariate analysis.

Risk factor	OR (95% CI)	*p* value
Major crimes	1.6 (0.8–3.4)	0.181
Hypertension	1.9 (0.9–3.9)	0.081
Smoking	1.7 (0.8–3.4)	0.131
Obesity	3.4 (1.5–7.7)	0.003

## Data Availability

The excel data used to support the findings of this study are restricted by the Ministry of Justice of Cameroon in order to protect prisoners privacy. Data are available from Sylvain Raoul Simeni Njonnou after authorization of the Ministry of Justice of Cameroon for researchers who meet the criteria for access to confidential data.

## Abbreviations

ADA: American Diabetes Association
CVRF: Cardiovascular risk factor
IDF: International Diabetes Federation
NCD: Noncommunicable diseases
SPSS: Statistical Package for the Social Sciences
USA: United States of America.
